# Clinical Cases in the Medical Wards of Mercy Hospital

**Published:** 1872-12-15

**Authors:** N. S. Davis


### CLINICAL CASES IN THE MEDICAL WARDS OF THE MERCY HOSPITAL.

SERVICE OF PROF. N. S. DAVIS.

*Clinic of Nov. 29th*, 1872.

Case I.— *Chronic Dysentery.* — The first case to which I will call your attention, is one that you examined fully in one of the clinics last week. He is a man, 40 years of age, and, as you will remember, had been suffering many weeks with chronic dysentery.

When you examined him before, he was much emaciated; pulse soft, quick and weak; skin relaxed and clammy to the feel; countenance depressed and anxious; with frequent passages of bloody serum, mixed with whitish flakes and some mucus. These passages were not only frequent, but accompanied and preceded by great pain and tenesmus.

Previous to his admission to the hospital and during the week preceding your examination of his case, he had taken quite a variety of remedies, with but little apparent influence over the progress of the disease. Owing to this fact, and the increasing exhaustion, as indicated by the soft, weak pulse and relaxed skin, he had been put upon the two following prescriptions:

| | | |
|---|---|---|
| ℞ | Ol. terebinth., | ʒ iii. |
| | Ol. wintergreen, | 30 gtts. |
| | Tinct. opii, | ʒ iv. |
| | Pulv. gum arabic, } aa | ʒ vi. |
| | White sugar, } | |
| Rub together and add | | |
| | Water, | ℥ iv. |

Mix, and take one teaspoonful every three hours.

| | | |
|---|---|---|
| ℞ | Strychnia, | 1 gr. |
| | Nit. acid, | ʒ i. |
| | Tinct. opii, | ʒ iv. |
| | Simple syrup, | ℥ ss. |
| | Water, | ℥ iiss. |

Mix, and take one teaspoonful in a tablespoonful of sweetened water, half way between the doses of the preceding prescription.

Directions were also given to have an enema containing one drachm of syrup of ipecac and half a drachm of tincture of opium in two ounces of water, administered evening and morning. For nourishment the patient took chiefly wheat flour and milk porridge. For four days after you saw him, he pursued this treatment without any material change, and it resulted in a decided improvement in all his symptoms. The discharges were reduced to three or four in the twenty-four hours, and contained very little appearance of blood or mucus, and were accompanied by no pain. But the strychnia and nitric acid mixture now began to irritate his stomach, and it was omitted, and the terebinthinate emulsion, given every two hours. The enemas were limited to one every evening. At the present time he is feeling quite well, and is having only one or two passages in the twenty-four hours. It will be necessary, however, to continue the emulsion and a careful regulation of his diet until the passages are not only reduced to one per day, but also assume a healthy, consistent character. How far ipecac adds to the efficacy of enemas in dysentery, I will not venture an opinion, simply because I have resorted to it in only a few cases. The first case in which I resorted to it, was that of a delicate lady who had been attacked with dysentery during childbed, and in whom it proved very obstinate. I tried faithfully for ten days to control the disease, but without any desirable progress. On the contrary, she was rapidly failing in strength and flesh, and her stomach had become so irritable, that it was difficult for her to retain anything.

In this condition I desisted from the exhibition of active remedies by the mouth, and directed that she should have an enema of ten grains of pulverized ipecac and one half grain of sulphate of morphia, in two ounces of water, every four hours, until the passages were controlled. This course was followed by the most decided relief and the rapid recovery of the patient.

Case II.—*Pleuro-Pneumonia.*—The next case is also one which you examined fully several days since. It was in the person of a man, aged thirty-five years, slender in form, and nervous in temperament. At the time of his admission into the hospital, he had been laboring under an acute attack of pleuro-pneumonia for about one week. At the time you first saw him, which was only two days after his admission, his face was dusky or leaden; pulse small, quick, and weak; extremities cool; breathing short, hurried, and much oppressed; stomach and bowels quiet, but much pain in the lower part of the left side of his chest. You will remember that he was expectorating a very large quantity of muco-purulent matter, mixed with dark blood, and the whole left side of the chest was moderately enlarged, the intercostal spaces full; complete dullness on percussion from the clavicle to the diaphragm, and silence on auscultation, while the right side appeared to be natural. The complete dullness on percussion, with enlargement of the side, rendered it certain that he had pleurisy with effusion, while the copious bloody expectoration was equally indicative of pneumonia, with copious exudation.

You will also remember that I then called your attention to the quantity and quality of the expectoration, in connection with the naturally slender or phthisical appearance of the patient, as justifying the apprehension that the pneumonic exudation, instead of undergoing re-absorption, would take on the degenerative process styled caseous degeneration, and end in extensive diffuse suppuration and death. To check this tendency, and at the same time mitigate the cough and pain in his side, I directed a blister on his side, and a teaspoonful of the following mixture every three hours:

| | | |
|---|---|---|
| ℞ | Hydrochlorate ammonia, | ʒ iii. |
| | Tart. ant. et pot., | 2 grs. |
| | Sulph. morph., | 3 grs. |
| | Syrup of liquorice, | ℥ iv. |

Mix; and half way between each of these doses, five grains of carbonate of ammonia, one of pulverized gum camphor, and one of sulphate of quinine, suspended in mucilage of gum arabic. Also, as much milk and animal broth for nourishment as his stomach would bear. He followed this treatment for five or six days, when your attention was called to him again. The pain had then disappeared from his side; the capillary circulation in the surface and the temperature of the extremities had improved; the cough was less severe; the expectoration still copious and puruloid, but nearly destitute of blood; the respiration better, but still short, and the physical signs derived from auscultation and percussion, the same as before.

I then told you we would continue to give him the anodyne and expectorant mixture every four hours, but instead of the carbonate of ammonia and camphor, we would give a small tablespoonful of a mixture of extract of malt, two parts, and compound syrup of hypophosphites, one part, hoping that the latter would be more efficient in lessening the tendency to suppuration, and in promoting the process of nutrition. He has continued that treatment until the present time, with a steady improvement in all his symptoms. He now coughs and expectorates but little; has a fair appetite and is steadily increasing in strength. The left side of the chest is diminished in size; a slight respiratory murmur can be heard over the upper and anterior part, but there is still general dullness on percussion. I shall advise a continuance of the same treatment.

Case III.—*Chronic Gastritis.*—The next patient before you is a man, about forty-five years of age, a laborer; his surface is white, as in spanemia; his pulse is soft and weak, but not more than eighty-five per minute; his bowels nearly natural; but his flesh is soft and moderately emaciated, and he has for several months complained of muscular weakness and great distress in the stomach after taking food, and frequent vomiting. The distress is sometimes a hard pain in the epigastrium, but more frequently a burning and sense of heaviness, immediately aggravated by taking food, which latter is often rejected by vomiting, accompanied with much acid mucus. And if the food is not rejected, its digestion is accompanied by much flatulency, with gaseous and sour eructations. His bowels are easily moved by laxatives, and his urine nearly natural. His age, and pallid appearance, and moderate loss of flesh, would suggest the probable existence of cancerous disease of the pyloric portion of the stomach. But in this form of disease, there is generally less flatulency, less acid or sourness, and less burning distress and vomiting immediately after taking food. In cancer of the pylorus, the distress after eating comes later, and the vomiting is more uniform, both in time after eating, and in being chiefly a thick, ropy mucus; more decided constipation, and in most cases presenting a hard tumor which can be felt in the lower part of the epigastrium, when the patient is on his back, with the thighs flexed sufficient to relax the abdominal muscles.

When cancerous disease exists at the cardiac orifice of the stomach, the patient generally feels a difficulty of deglutition, or rather the food seems to stop and create distress before it fairly enters the stomach.

After a careful comparison of the symptoms of chronic inflammation of the mucous membranes of the stomach, and cancerous disease, and being unable to find any hardness or tumor in the region of the pylorus, I am constrained to regard the case before you as one of chronic gastritis. With this view of his case, when he first came under my care, about four weeks since, his diet was limited to small quantities of the more bland articles, and he was given a teaspoonful of the following solution before each meal, and a powder of six grains of sub-nitrate of bismuth and one-quarter of a grain of sulphate of morphia, at bed-time.

| | | |
|---|---|---|
| ℞ | Carbolic acid crystal., | 6 grs. |
| | Glycerine, | ℥ ss. |
| | Tinct. gelsemin, | ʒ iii. |
| | Camph. tinct. opii, | ℥ i. |
| | Water, | ℥ ii. |

We continued this treatment, with an occasional laxative pill at night, for three weeks; and after the first four or five days, he was so much relieved that he had very little flatulency or pain in the stomach; did not reject his food by vomiting more than once or twice per week. About one week since, however, he began to complain of a return of all of his old symptoms, more especially of the flatulency and acid eructations. I then discontinued the carbolic acid mixture, and gave in its place the following:

℞ Sulphite of soda, ʒ iv.

Tinct. belladonna, ʒ iv.

Simple syrup, ʒ iv.

Mint Water, ℥ iii.

Mix. Give one teaspoonful before each meal, and continue the bismuth powder at bed-time. This has again entirely relieved the irritation and symptoms of indigestion. But to prevent constipation, a compound rhubarb pill must be given at night, instead of the bismuth, at least a part of the time.

